# Secondary Interface Echoes Suppression for Immersion Ultrasonic Imaging Based on Phase Circular Statistics Vector

**DOI:** 10.3390/s23031081

**Published:** 2023-01-17

**Authors:** Ming Chen, Zhenghui Xiong, Yan Jing, Xi He, Qingru Kong, Yao Chen

**Affiliations:** 1 Key Laboratory of Non-Destructive Testing Technology, Ministry of Education, Nanchang Hangkong University, Nanchang 330063, China; 2 AECC Aviation Power Co., Ltd., Xi’an 710021, China

**Keywords:** phase circular statistics vector, suppress, total focusing method, secondary interface echoes, immersion

## Abstract

Immersion ultrasonic phased array imaging technology offers great advantages, particularly in coupling and automatic detection of industrial non-destructive testing (NDT). To suppress the influence of secondary interface echoes in the immersion ultrasonic phased array imaging, a novel phase circular statistics vector (PCSV) weighting method is proposed in this paper. Firstly, the PCSV factor matrix is established according to the phase consistency of the echo signals. Secondly, due to the higher phase coherence of the defect echo, the PCSV factor matrix is used to weight the TFM image to suppress the secondary interface echo. The result shows the secondary interface echoes are effectively suppressed in the total focusing method (TFM) image on a 0~40 dB scale. It is also shown that PCSV weighting could not only suppress the secondary interface echoes but also improved the image quality in terms of SNR and lateral resolution by comparing with traditional TFM.

## 1. Introduction

Ultrasonic phased array imaging techniques, such as the synthetic aperture focusing technique (SAFT) and total focusing method (TFM), have been widely used in non-destructive testing (NDT) [1,2,3,4,5]. In practice, to ensure constant acoustic coupling, the immersion phased array imaging technology is often used in NDT inspection [6,7]. It has remarkable advantages such as protecting the transducer from frictional wear, accommodating object shape variations, and realizing the automated test [8]. Thus, the immersion phased array imaging technology can easily improve the coupling quality and inspection efficiency.

In contacted nondestructive testing cases, the influence of additional coupling medium can often be ignored. However, in the immersion case, a two-layered system is formed because of the different acoustic impedance between the water and the object [9]. Under this circumstance, when the coupled water layer has a certain thickness, the reflection will disturb the object detection, so the influence of the water layer cannot be ignored. In practical immersion testing, the presence of multiple interface echoes may interfere and overlap with the defect echo or be misinterpreted as the primary echo [10,11]. Therefore, how to deal with the multiple interface echoes are worth under consideration for the immersion phased array imaging technology.

As early as in the field of seismic imaging and marine geophysical prospecting, multiple interface echo suppression has been widely concerned, especially in data processing and imaging applications [12,13]. From previous studies on seismic data, it has been proven that the desired primary interface echo and the undesired multiple interface echoes are different in many properties, such as phase characteristics, apparent velocity, frequency component, periodicity, and so on [14,15]. Hence, based on the above difference characteristics, one of the most important methods to suppress multiple interface echoes is filtering or weighting. This method can suppress the sea surface multiple reflection in marine geophysical exploration and the interlayer multiple reflection in seismic data interpretation. Looking back at the immersion ultrasonic phased array imaging in industrial NDT, it is found that the effect of undesired multiple reflections at the water-object interface is very similar to that of interlayer multiple reflections. If the difference between the undesired echoes and desired echoes could be used to design a good filter, it is expected to solve the impact of multiple reflections of water-object interface in NDT.

In recent years, a technique termed phase coherence imaging (PCI) which is based on the phase consistency difference between defects and undesired echoes has developed [16,17]. To suppress undesired echoes, the PCI technique makes use of a two-step imaging strategy. Firstly, the PCSV factor matrix is established according to the phase consistency of the echo signals. Secondly, due to the higher phase coherence of the defect echo, the PCSV factor matrix is used to weight the TFM image to suppress the secondary interface echo. Theoretically, PCI could suppress all undesired echoes. At present, PCI has been proven to suppress undesired noises through phase consistency differences [18,19]. Furthermore, it also could improve resolution by suppressing the undesired echoes around the defect [20,21]. Therefore, the PCI technique offers a potential solution for suppressing the multiple interface echo.

To suppress the multiple interface echo, a novel phase coherence factor, phase circular statistics vector (PCSV) has been proposed in this work. To verify the validity of the method, the PCSV was used to weight the TFM image with immersion. Next, two things have been carried out for investigating the suppression effect of the interface echo between the additional coupling medium layer and the object. The first thing to do is to verify that the value of the PCSV can distinguish and reveal the difference of phase consistency between the defect echo and multiple interface echoes from the multiple wave reflection between probe and the water-object interface. The second thing to do is to investigate the suppression effect and image quality by comparing before and after PCSV weighting.

## 2. Theory

### 2.1. Total Focusing Method in the Layered Object

The TFM imaging technique is generally a posteriori synthetic focusing [22] of full matrix capture (FMC) datasets acquired with a transducer array. An *N*-element array transducer performs a single-element for transmission and full elements for reception under test. If (*x_e_*, *z_e_*) and (*x_r_*, *z_r_*) are, respectively, the element position used for transmission and reception, the FMC dataset consists of a set of N*N A-Scan signals s*_er_*(*t*). Both indexes *e* and *r* denote a pair of transmitter-receiver.

**Figure 1:**
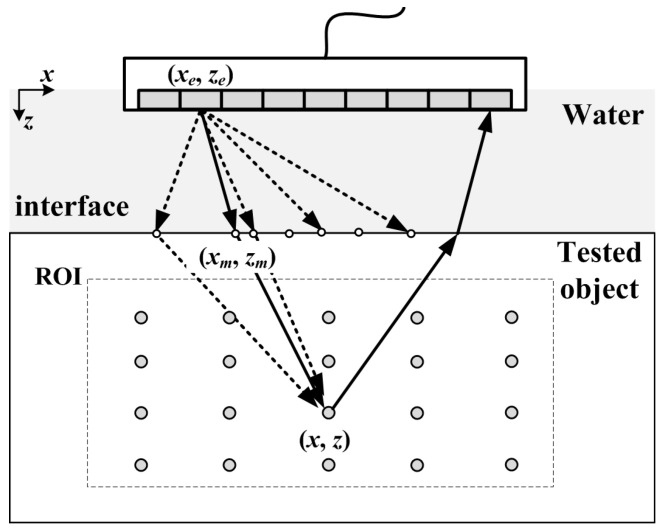
ROI of water-object material.

When in water immersion condition, ultrasonic signals emitted by element (*x_e_*, *z_e_*) will be refracted at the water-component interface, as shown in Figure 1. The reflected echo from the scattered point (*x*, *z*) is also refracted by the interface and then received by the element (*x_r_*, *z_r_*). Compared with the single-layer medium, the propagation path of sound waves in the double-layer medium will change in the immersed system. The distances between the scattered point (*x*, *z*), (*x_e_*, *z_e_*), and (*x_r_*, *z_r_*) are no longer the length of the straight line segment between these points, but the sum of the broken line segments in each layer of media.

For each scattered point (*x*, *z*) in a region of interest (ROI), the TFM algorithm needs to compute the theoretical times of flight (TOF) corresponding to the propagation time. According to Fermat’s minimum time theorem, the path that wave travels is the one that takes the least time. If there are B*M pixels in ROI, it is assumed that there are *M* virtual refraction points on the water-object interface, as shown in white dots on the interface in Figure 1.

As shown in Figure 1, (*x_m_*, *z_m_*) represents the coordinates of any refraction point calculated on the water-aluminum interface, which corresponds to the pixel coordinates on the interface. Next, the propagation time between *t_em_* the *e*th transmitter and the scattered point (*x*, *z*) through the refraction point (*x_m_*, *z_m_*) is:

(1) $$t_{em}=\frac{\sqrt{{\left(x_{e}-x_{m}\right)}^{2}+{\left(z_{e}-z_{m}\right)}^{2}}}{c_{1}}+\frac{\sqrt{{\left(x_{m}-x\right)}^{2}+{\left(z_{m}-z\right)}^{2}}}{c_{2}}$$

where *c*_1_ and *c*_2_, respectively, represent the velocity of the water and the object layer. Next, the possible TOF through the interface during the wave propagation can be calculated according to Equation (1). By taking the minimum value of all TOF calculation results, the shortest propagation time *t_T_* of the *e*th transmitter and the scattered point (*x*, *z*) through the refraction point (*x_m_*, *z_m_*) can be obtained:

(2) $$t_{T}=\min\left\{t_{e1},t_{e2},\ldots ,t_{em},\ldots ,t_{eM}\right\}$$

Similarly, the shortest propagation time *t_R_* of the *r*th receiver and the scattered point (*x*, *z*) through the refraction point (*x_n_*, *z_n_*) is:

(3) $$t_{R}=\min\left\{t_{r1},t_{r2},\ldots ,t_{rn},\ldots ,t_{rM}\right\}$$

where 1 ≤ *m*, *n* ≤ *M*. In that way, the total travel time can be evaluated numerically by *t_er_* = *t_T_* + *t_R_*.

The algorithm then requires the calculation of N*N times of flight for each pixel. The image obtained by TFM is achieved by summing all the signal amplitudes of the return wave extracted at the time *t* = *t_er_*, which can be expressed as:

(4) $$I_{TFM}(x,z)=\sum_{e=1}^{N}\sum_{r=1}^{N}s_{er}(t_{er})$$

where *s_er_*(*t_er_*) is the signal amplitude of scatter (*x*, *z*) received by element *r* from the transmit element *e*.

### 2.2. Phase Circular Statistics Vector Weighting Methodology

The proposed methodology to remove the multiple interface echo from the multiple wave reflection between probe and the water-object interface was to combine with Phase Coherence Imaging (PCI). The regular TFM process maps the amplitude of aperture data at each pixel in the image scene, whereas PCI maps the phase distribution of the aperture data at each pixel location.

**Figure 2:**
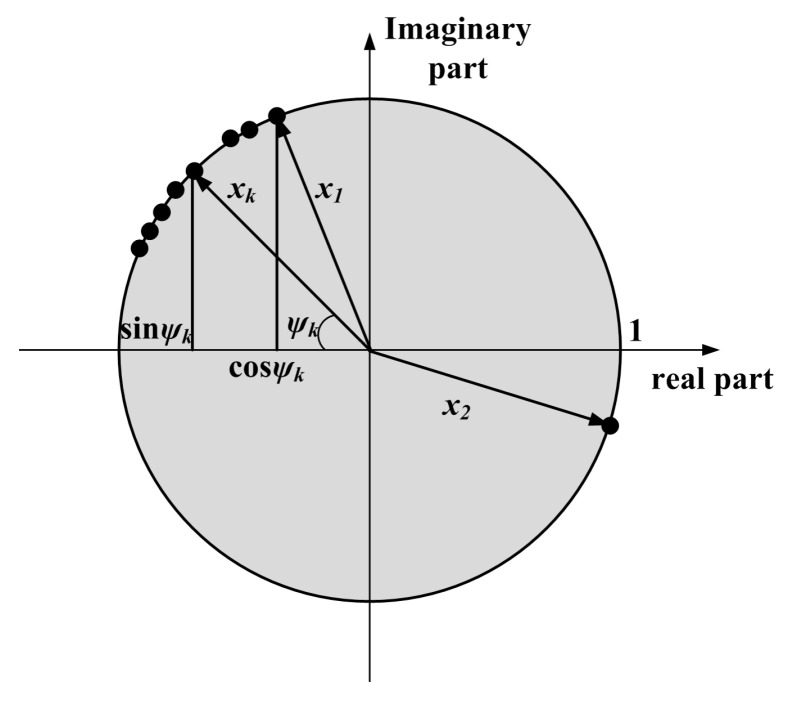
The representation of circular vector on the complex plane.

Circular statistics is a statistical analysis method in which the phase angle value *ψ* is used as the sample points [23,24,25]. The sample points are discretely distributed on the unit circle, as shown in Figure 2. The vectors (cos*ψ*, sin*ψ*) *ψ* are seen as the characterization of the sample point on the complex plane. The value of the resultant vector can represent the consistency of the phase distribution. When the value is larger, it indicates that the phase distribution is more consistent, and the sample points are closer to a specific direction. Conversely, it indicates that the consistency of phase distribution is low.

If *N* sample points are distributed on the unit circle, the average resultant vector *X* on the complex plane can be obtained by the vectorial resultant theorem and the Euler formula transformation [26]:

(5) $$X=\frac{1}{N}\sum_{k=1}^{N}e^{i\psi_{k}}=\frac{1}{N}\sum_{k=1}^{N}(\cos\psi_{k}+i\sin\psi_{k})=a+ib$$

where *i* is an imaginary unit, *ψ_k_* is the phase angle of the *k*th sample point, the *a* and *b* represent the real and imaginary parts of the resultant vector, and the expression can be written as:

(6) $$a=\frac{1}{N}\sum_{k=1}^{N}\cos\psi_{k},\;b=\frac{1}{N}\sum_{k=1}^{N}\sin\psi_{k}$$

According to Equations (5) and (6), the length of the average resultant vector is:

(7) $$R={\left(a^{2}+b^{2}\right)}^{1/2}$$

In Equation (7), the length value *R* is average resultant vector ranges among [0, 1]. When *R* = 1, all sample points are highly consistent, and all the sample points are in the same direction. When *R* tends to be 1, it indicates that the sample points are gathered around the average direction. On the contrary, the smaller the length value, the lower the coherence of the sample points is.

If set the collected FMC signals as *s*, the instantaneous phase *φ*(*t*) of *s* can be extracted by Hilbert transform in Equation (8):

(8) $$\varphi (x,z,t)=\arctan\frac{H(s)}{s}$$

where *H*(·) is the Hilbert transform of the signal *s*. Each of these angles determines each vector on the unit circle of the scattered point (*x*, *z*) with different transmitter-receiver pairs.

According to Equation (8), the *φ*(*t*) obtained reflects the original signal and its Hilbert transform, which does not lose any useful information. Next, the matrix cos*φ* and sin*φ* of the instantaneous phase can be easily constructed. It represents the real and imaginary parts of the phase information in the Euler formula, respectively. The cosine and sine matrix image is determined by using Equations (9) and (10):

(9) $$I_{\cos}(x,z)=\sum_{e=1}^{N}\sum_{r=1}^{N}\cos(\varphi_{er}(t_{er}))$$

(10) $$I_{\sin}(x,z)=\sum_{e=1}^{N}\sum_{r=1}^{N}\sin(\varphi_{er}(t_{er}))\;$$

All the real and imaginary parts of the phase from the scattered point are summed after the above two steps. According to Equations (5)–(7), it is obvious that a phase circular statistics vector (PCSV) can be constructed which has all the information of the phase. It is defined as:

(11) $$R_{PCSV}=\sqrt{(I_{\cos}^{2}+I_{\sin}^{2})/N^{2}}\;$$

where 0 < *R* < 1.

Finally, the reconstructed amplitude matrix *I_TFM_* is weighted with phase circular statistics vector, and the expression of the focused imaging is obtained as follows:

(12) $$I_{PCSV-TFM}(x,z)=R_{PCSV}\cdot I_{TFM}\;$$

## 3. Experiments

**Figure 3:**
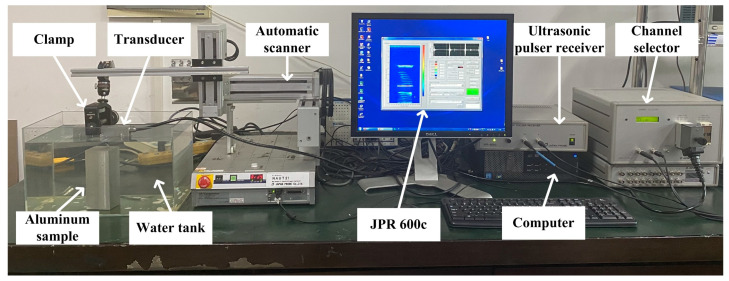
Data acquisition platform.

The experimental data signals were used to validate the effectiveness of the proposed method in real conditions. The water-immersion ultrasonic testing system for the signal acquisition was as shown in Figure 3. The liner array with *N* = 128 elements, *f* = 5 MHz, and pitch *d* = 1 mm (Guangzhou Doppler Electronic Technology Co., Ltd., Guangzhou, China). The JPR 600C as an ultrasonic pulser receiver (Nippon Probe Corporation, Yokohama, Japan) with 128 active channels was used to generate the ultrasound wave and to capture the datasets. A 20 MHz sampling frequency was used. The received experimental data signals were transferred to the computer and further processing was carried out off-line using MATLAB. Acquisition and processing parameters for the experiment are listed in Table 1.

**Figure 4:**
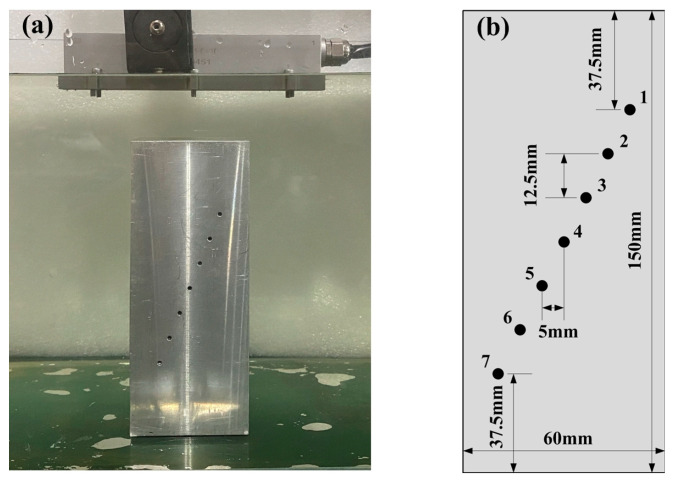
The array probe and sample for the experiment: (**a**) the probe and sample; (**b**) the defect of the sample.

The test aluminum sample with artificial defects used in the experiments can be seen in Figure 4. To capture the FMC matrix a linear-array transducer was directly placed above the sample. The relative position of the probe and the aluminum sample is shown in Figure 4a. The test aluminum sample as shown in Figure 4b with a length of 150 mm was adopted in this experiment. The sample is machined with a series of diameter 2 mm side holes at different depths. The lateral and vertical spacing between these reflectors is 5 mm and 12.5 mm. The acquired data set allows imaging of 7 reflectors with a depth range from 37.5 mm to 112.5 mm. These holes are numbered 1–7 from top to bottom. 

To simulate the influence of secondary interface echoes on No. 4–7 detects, the thickness of the water layer is adjusted to 17 mm–25 mm by controlling the probe height. The purpose of this simulation is to research the secondary interface echo suppression for different depths. In the experiment, the FMC datasets contains the interface and defect echoes were collected by data acquisition platform. These datasets were all stored in the computer and imported into MATLAB software. Based on Equations (1)–(12), TFM and PCSV-TFM images are compared to analyze the effectiveness of the proposed method. The numerical calculation software version is MATLAB 2018a. The CPU used in the test was Intel core i7-6700 with the main frequency of 3.4 GHz.

## 4. Results and Discussion

### 4.1. Comparison of the Consistency of the Phase Distribution

**Figure 5:**
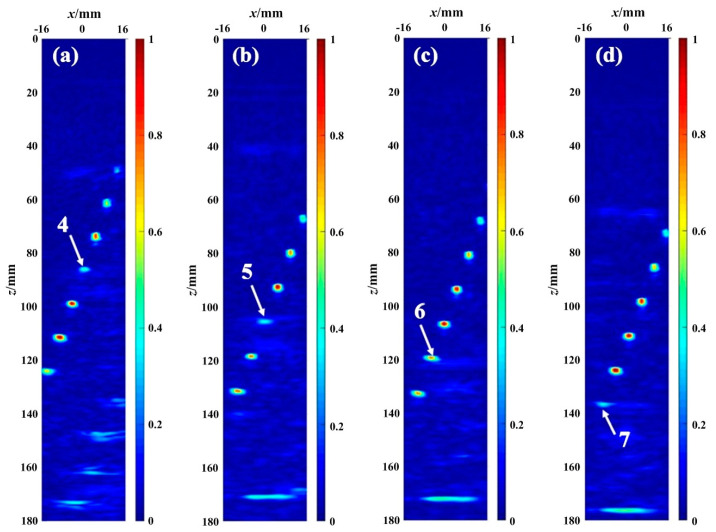
The PCSV factor images of different defects covered by the interface second echo: (**a**) the fourth defect covered; (**b**) the fifth defect covered; (**c**) the sixth defect covered; (**d**) the seventh defect covered.

Figure 5 is the graphical display of the PCSV factor matrix calculated based on Equations (8)–(11). Figure 5, respectively, shows the PCSV factor when (a) the fourth, (b) the fifth, (c) the sixth, and (d) the seventh defects are covered by the secondary interface echo. During data collection, to ensure that the defect covered by interface echoes was in the imaging area, the defects in other locations may be out of the region. For example, the first defect in Figure 5b–d is not in the ROI. The PCSV factor matrix images were all normalized to a 0 to 1 scale. The value of each pixel in the figure represents the phase consistency of the received signal at that point after the delay process. Figure 5 shows the echoes of defect have the higher phase consistency than the interface echoes.

The red in the color bar is taken as the standard. The closer the defect color is to red, the greater the value of the PCSV factor. That is, the higher the phase consistency at the defect. On the contrary, the closer the defect color is to blue, the lower the phase consistency at the defect.

As can be seen from Figure 5, the color of the PCSV factor at the defect location is brighter than the surrounding pixels. It indicates that the phase consistency degree from a perfectly focused defect is usually high. Furthermore, by comparing the color of the PCSV factor at defect location, it can be seen that the color of the PCSV factor of interface echo is more shallow and almost invisible in the PCSV factor figures. It shows that the PCSV factor of the defect echo covered is higher than the interface echo but is inferior to other defects uncovered. This because the interference of interface echo weakens the phase consistency degree of the covered defect.

From the above results, although the phase consistency degree of the defect covered is weakened, the difference between the secondary interface echo and the defect echo could still be characterized by using the PCSV factor. In the PCSV factor, the phase consistency degree of the interface echo is lower than that of the defect. Hence, the PCSV factor has the potential to distinguish the interface echo and the defect echo.

### 4.2. The TFM Imaging Results after Weighting by PCSV Factor

According to Figure 5, the PCSV factor of the secondary interface echo is not completely equal to 0. The PCSV of some undesired echoes still exists. This may result in the retention of some non-defect echoes with high amplitude. To further suppress other undesired noises and preserve the contrast of the PCSV difference between the defect and the interface echo, the power transformation from the image processing field is introduced [27,28]. The exponential PCSV can be expressed as:

(13) $${\left(R_{pcsv}\right)}^{\sigma }={\left(\sqrt{(I_{\cos}^{2}+I_{\sin}^{2})/N^{2}}\right)}^{\sigma }\;$$

where *σ* is the power exponent of the phase factor PCSV. If *σ =* 0, the value is equal to 1 at any point, and no weighting is applied. If *σ >* 0, it is a variable parameter that controls the sensitivity of the weighting factor. when 0< *σ* <1, the effect of the PCSV factor is weakened. When *σ* > 1, the effect of the PCSV is enhanced. However, echoes from the defect would be suppressed significantly at a larger value *σ* according to the previous work in literature [27]. To achieve a compromise inhibition, the value of *σ* is 2 in this experiment.

**Figure 6:**
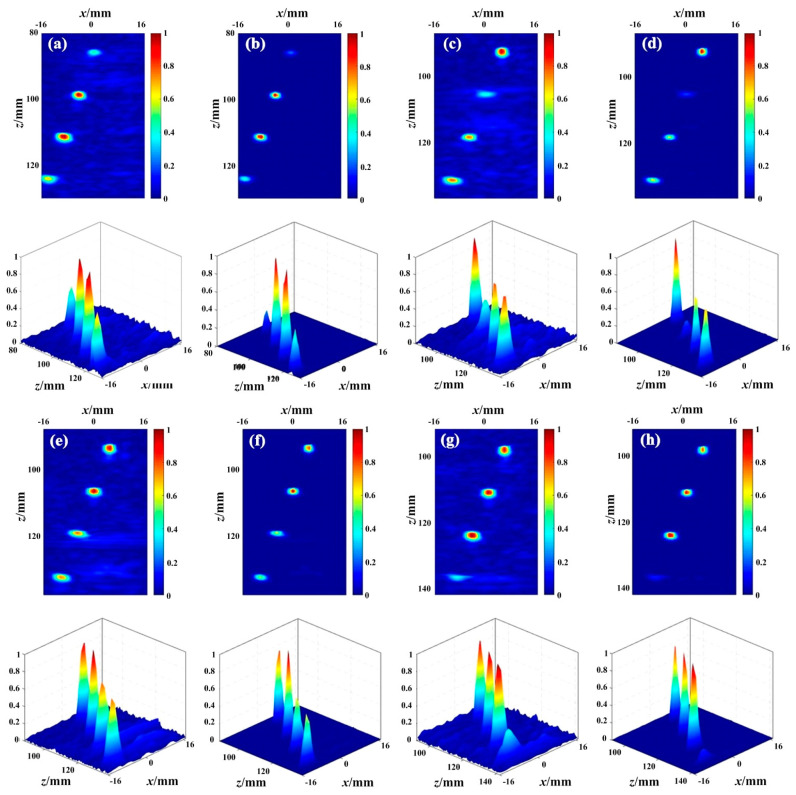
The PCSV and (PCSV) σ factors of different defects covered by the interface echo: PCSV and its 3D display of (**a**) the fourth defect covered; (**c**) the fifth defect covered; (**e**) the sixth defect covered; (**g**) the seventh defect covered; and PCSV with *σ* = 2 and its 3D display of (**b**) the fourth defect covered; (**d**) the fifth defect covered; (**f**) the sixth defect covered; (**h**) the seventh defect covered.

The local images of PCSV (*σ =* 1) and the exponential PCSV with *σ =* 2 are shown in Figure 6. Each local image contains the imaging region from the fourth defect to the seventh defect. The local size is 50 mm × 32 mm. To compare the PCSV variation more clearly, The 3D display of each image follows. Figure 6a,b is the result of the fourth defect covered by interface interference. Figure 6c,d is the result of the fifth defect covered by interface interference. Figure 6e,f is the result of the sixth defect covered. Figure 6g,h are the result of the seventh defect covered by interface interference.

In Figure 6a, the values of the PCSV factor of the defects are about 0.4, 0.9, 0.9, and 0.6. The PCSV of the undesired echo is about 0.1. Compared with the PCSV factor in Figure 6a, the values of defects in Figure 6b are about 0.2, 0.8, 0.8, and 0.4. The values of undesired echoes are almost close to 0. The ratio of the PCSV between the interface and the defect covered is increased. It is expected to enhance the effect of the PCSV factor and suppress more interface echo. The results of defects covered at other depths are similar to those of the fourth defect covered in Figure 6a,b.

Consequently, by changing the *σ* value according to actual demands, the effect of PCSV will be controlled. With an increase in *σ*, the undesired echoes are suppressed and the width of the defects is reduced. Therefore, selecting a reasonable *σ* value for PCSV can effectively reduce the factor value of interface echo. The difference between secondary interface echo and defect echo could be further enlarged. It is expected that after weighting with TFM, no secondary interface echoes could be seen within the same image display range. The defects covered also could be clearly seen.

**Figure 7:**
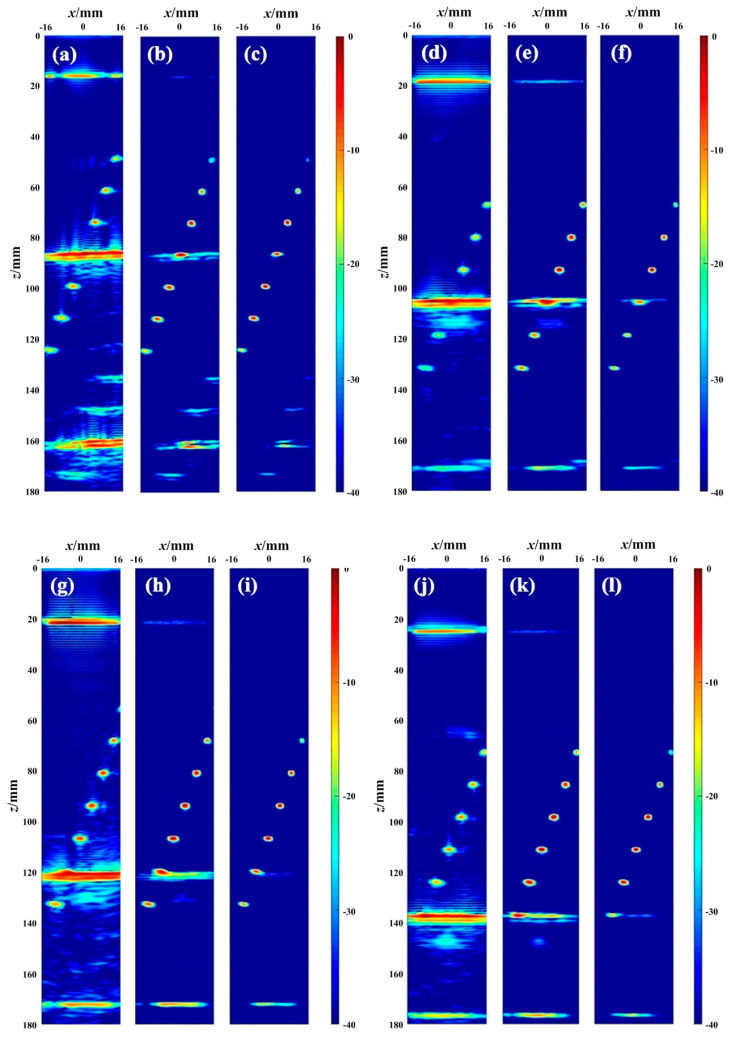
The image results of the different defects covered by the interface echo: (**a**) TFM; (**b**) PCSV-TFM; (**c**) PCSV-TFM with the power exponent *σ* = 2; of the fourth defect covered; (**d**) TFM; (**e**) PCSV-TFM; (**f**) PCSV-TFM with the power exponent *σ* = 2; of the fifth defect covered; (**g**) TFM; (**h**) PCSV-TFM; (**i**) PCSV-TFM with the power exponent *σ* = 2; of the sixth defect covered; (**j**) TFM; (**k**) PCSV-TFM; (**l**) PCSV-TFM with the power exponent *σ* = 2; of the seventh defect covered.

By using the PCSV weighting process, a series of images of TFM, PCSV-TFM, and PCSV-TFM with *σ* = 2 is obtained. These images are shown in Figure 7. The four groups, respectively, show the fourth, fifth, sixth, and seventh defects covered by the interface echo at different depth. Each group from left to right is as follows: (a) TFM amplitude images (b) TFM images multiplied by the PCSV factor (c) TFM images multiplied by the PCSV factor with *σ* = 2. All reconstructed images were displayed in decibel and plotted on a −40 to 0 dB scale.

Even without detailed analysis, the differences between the images before and after PCSV weighting are visible in these figures. It can be seen from Figure 7a that when the water layer is 17 mm, the fourth defect at depth 75 mm in the object has been mixed by the secondary interface echo in TFM. Since the width of the water object interface is much larger than the defect width, the echo range and amplitude are larger than the defect. If the position of the defect is unknown in advance under the practical detection condition, it is difficult to accurately determine whether there is a defect based on the TFM image. However, the locations of other defects are well revealed in the TFM image. 

Figure 7b shows the result of the TFM image weighted by the PCSV factor (TFM-PCSV). It can be seen from the figure that the amplitude of the secondary interface echo decreases to some extent. The defect covered has clearer visualization than TFM now. Furthermore, comparing with TFM images, the amplitudes of other defects after this process are still well retained even higher. The lateral width of defects is also a little narrower. However, due to the amplitude of the secondary echo in TFM is already very high and all PCSV factors are greater than 0, the interface echo around the defect is suppressed.

Figure 7c is the PCSV-TFM image with *σ* = 2. After weighted with TFM, Figure 7c shows that there is almost no interface echo at the depth of the defect covered under the −40 dB dynamic range. Although the value of the fourth defect in Figure 4b is low, the defect echo and secondary interface echo also could be separated due to the PCSV of interface echo is close to 0. Comparing with Figure 7b, the edge of the defect covered is more distinct in Figure 7c. It indicates that the contrast between defect echo and interface echo could be further improved by the PCSV factor after power transformation. 

When the water layer thickness is 20 mm, 23 mm, 26 mm, the secondary echo of the interface just, respectively, covers the fifth, the sixth, and the seventh defect, as observed in other figures in Figure 7. They all validate the results of the above analysis of the case of the fourth defect covered. 

Thus, by weighting TFM with the PCSV factor, the secondary interface echo with high amplitude in TFM was reduced whereas the defect echo was retained. In this way, the interference of secondary interface echo on the defect echo could be suppressed, and the covered defects could be visualization. If TFM weighted with PCSV after reasonable power transformation, the defect covered by secondary interface echo may be more easily identified and the secondary interface echoes may be invisible in −40 dB dynamic range.

### 4.3. The Analysis of Image Quality

**Figure 8:**
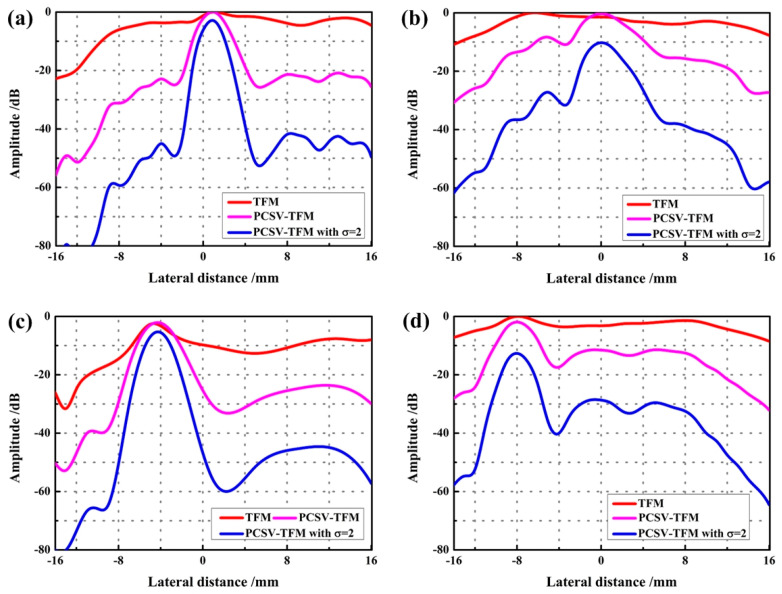
The lateral variations at the depth of (**a**) the fourth defect covered; (**b**) the fifth defect covered; (**c**) the sixth defect covered; (**d**) the seventh defect covered; by using TFM, PCSV-TFM, and PCSV-TFM with power exponent *σ* = 2.

To further analyze the suppression effect of interface echo after PCSV weighting, the lateral amplitude variations at the depth of defects covered in Figure 7 were extracted. These curves are displayed in Figure 8. Each line represents the variation according to the *x*-direction. These images, respectively, are (a) the fourth, (b) the fifth, (c) the sixth, and (d) the seventh defects are covered.

Looking at the direction of the curve, the TFM curve is almost horizontal. Statistically speaking, the maximum amplitudes of the main lobe in TFM are been about 0 dB, −2 dB, −3 dB, and 0 dB when the side lobe are been about −4 dB, 0 dB, −9 dB, and −3 dB. It is only a 2~6 dB difference between the defect and the secondary interface echo in TFM. Such a small amplitude difference is not sufficient to distinguish defects from interface echoes at 0~−40 dB. In Figure 8c, the difference is slightly obvious. This is because the position of the interface echo is below the defect and does not completely cover the defect in the original data.

Comparing with TFM curves, the main lobe of curves of the defect is high whereas the grating lobe is low after PCSV weighting. In Figure 8, after PCSV processing, the amplitude of interface echo could be reduced by about 10~20 dB, while in PCSV-TFM with *σ* = 2, the amplitude of interface echo could be reduced to about 30~40 dB. It indicates that the difference between interface echo and the defect in PCSV-TFM and the PCSV-TFM with *σ* = 2 is evident. Defects are easier to visualize at 0~40 dB. Moreover, it can be observed that the TFM and PCSV-TFM have almost similar peak levels of echoes from the defect. However, the PCSV-TFM with *σ* = 2 is slightly lower than them. This shows that PCSV after power transformation will bring a certain amplitude loss.

**Figure 9:**
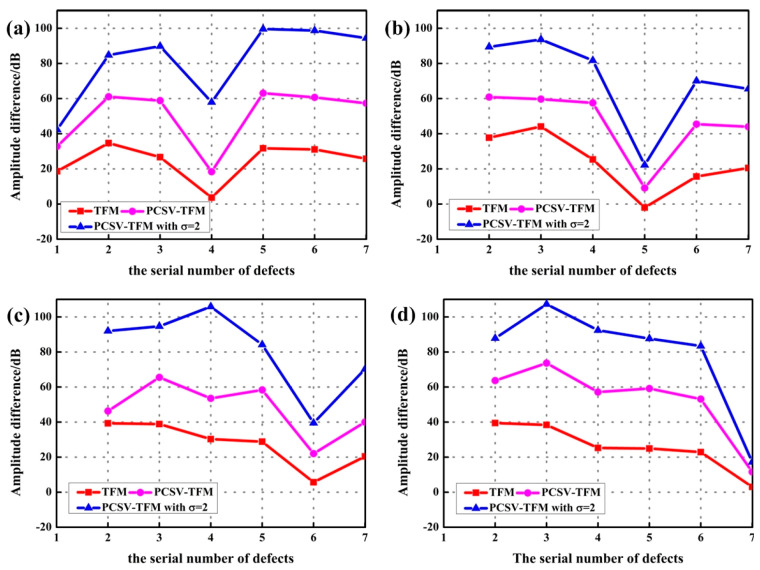
The amplitude difference between defects and the undesired echo around them at the depth of (**a**) the fourth defect covered; (**b**) the fifth defect covered; (**c**) the sixth defect covered; (**d**) the seventh defect covered; by using TFM, PCSV-TFM, and PCSV-TFM with power exponent *σ* = 2.

**Figure 10:**
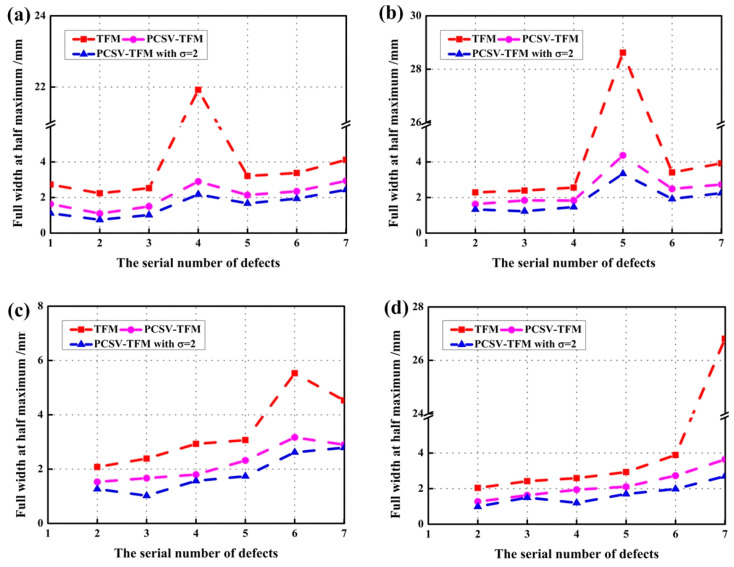
The full width at half maximum at the depth of (**a**) the fourth defect covered; (**b**) the fifth defect covered; (**c**) the sixth defect covered; (**d**) the seventh defect covered; by using TFM, PCSV-TFM, and PCSV-TFM with power exponent *σ* = 2.

For the quantitative measurement of imaging quality, the signal-to-noise ratio (SNR) and the lateral spatial resolution were measured. The SNR was evaluated by estimating the specific amplitude difference between defects and undesired echoes around them. The lateral spatial resolution was evaluated by estimating the full width at half maximum (FWMH) along the *x*-axis of the defect echo. The higher the difference is, the better the SNR of the image is. The lower the FWHM is, the better the lateral resolution of the defect is. The results of all defects in each figure were calculated and showed in Figure 9 and Figure 10, respectively.

According to Figure 9, the PCSV-TFM and PCSV-TFM with *σ* = 2 acquire higher difference values than TFM no matter where the defect depth is. In addition, the PCSV-TFM with *σ* = 2 is the highest where the difference value could be up to 60 dB at the defect covered and to 100 dB at the defects uncovered. It illustrates that using PCSV weighting could not only suppress the influence of interface echo but also improved the SNR level between defects and undesired echoes.

However, when the defect is covered by the interface echoes, the amplitude difference is about 40~60 dB lower than that of the defects uncovered even if it is after PCSV weighting. This indicates that the interference of interface echo has a great impact on the covered defects and could not be ignored in the imaging process. The amplitude difference of other uncovered defects is relatively flat.

Figure 10 displays the full width at half maximum of different algorithms at different depths of the defect covered [29]. In Figure 10, the FWHM of the defect covered in TFM is approximately 22 mm, 29 mm, 5.5 mm, and 27 mm. These values are well beyond the diameter of the defect. This indicates that there is a high interface echo around the defect and the lateral resolution of the defect could not be evaluated in TFM. Comparing with TFM, the FWHM of defects is 1.5~3 mm in PCSV-TFM and 1~2 mm in PCSV-TFM with *σ* = 2. It can be seen the lateral resolution after PCSV weighting is higher and the PCSV-TFM with *σ* = 2 is the highest. Therefore, the PCSV weighting could guarantee better lateral resolution than TFM over a larger dynamic range when the secondary interface echoes are suppressed. Similar to the result of SNR, when the defect is covered by interface echo, the FWHM of the defect basically higher than that of other uncovered defects. The interface echoes also affect the lateral resolution of the covered defects.

Evaluation of image quality revealed that TFM with PCSV weighting yielded better defect visualization than traditional TFM. However, when the defect is covered by the secondary interface echo, the phase consistency degree of the defect is weakened. Compared with other defects not covered, the amplitude of defects covered is lower after PCSV weighting. The signal-to-noise ratio and lateral resolution are also inferior to defects not covered. The interference of secondary interface echo will hardly affect defects not covered. Although the imaging quality of the defect covered is not as good as that of the defect not covered, that will not affect the identification of the defect covered.

## 5. Conclusions

In this paper, the PCSV factor is proposed as a weighting method to suppress the influence of secondary interface echoes in the phased array imaging with immersion systems. The difference in the phase consistency degree between the secondary interface echo and the defect echo can be differentiated by the PCSV factor. In the PCSV factor, the phase consistency degree of interface echo is lower than that of the defect echo. The TFM weighted by PCSV could effectively suppress the impact of the defect covered by the secondary interface echo. Due to the weakening effect of the secondary interface echo at the phase consistency of the defect echo, the imaging quality of the defect covered after PCSV weighted processing is lower than that of the defects not covered. However, the defect covered is still able to be recognized. Therefore, by PCSV weighting implementation, the suppression of secondary interface echo and the improvement of defect visualization could be realized simultaneously for the immersion system.

## Figures and Tables

**Table 1:** Acquisition and processing parameters for the experiment.

Parameters	Value
Number of elements to use	32
Element width	0.9 mm
Element pitch	1 mm
Center frequency	5 MHz
Sampling frequency	20 MHz
Excitation voltage	100 V
Speed of sound in water	1480 m/s
Speed of sound in aluminum	6300 m/s

## Data Availability

Not applicable.
